# Intra-venous bevacizumab in hereditary hemorrhagic telangiectasia (HHT): A retrospective study of 46 patients

**DOI:** 10.1371/journal.pone.0188943

**Published:** 2017-11-30

**Authors:** Alexandre Guilhem, Anne-Emmanuelle Fargeton, Anne-Claire Simon, Pierre Duffau, Jean-Robert Harle, Christian Lavigne, Marie-France Carette, Olivier Bletry, Pierre Kaminsky, Vanessa Leguy, Nathalie Lerolle, Dominique Roux, Marc Lambert, Thierry Chinet, Delphine Bonnet, Sophie Dupuis-Girod, Sophie Rivière

**Affiliations:** 1 Médecine Interne - Maladies Multi-Organiques, Hôpital Saint Eloi, Montpellier, France; 2 Service de Génétique - Centre de Référence National Maladie de Rendu-Osler, Hôpital Femme-Mère-Enfant, Bron, France; 3 Service de Pneumologie, CHU de Poitiers, Poitiers, France; 4 Service de Médecine Interne et Immunologie Clinique, Hôpital Saint-André, Bordeaux, France; 5 Département de Médecine Interne, Hôpital de la Timone, Marseille, France; 6 Médecine Interne et Maladies Vasculaires, CHU d’Angers, Angers, France; 7 Service de Radiologie, Hôpital Tenon, Paris, France; 8 Service de Médecine Interne, Hôpital Foch, Suresnes, France; 9 Service de Médecine Interne, Hôpital de Brabois, Vandoeuvre-lès-Nancy, France; 10 Service de Médecine Interne et Immunologie Clinique, Hôpital François Mitterrand, Dijon, France; 11 Service de Médecine Interne, Hôpital Bicêtre, Paris, France; 12 Service de Médecine, Centre hospitalier, Ambert, France; 13 Service de Médecine Interne, Hôpital Huriez, Lille, France; 14 Service de Pneumologie, Hôpital Ambroise Paré, Boulogne-Billancourt, France; 15 Service de Médecine Interne du Pôle digestif, CHU Purpan, Toulouse, France; Stanford University, UNITED STATES

## Abstract

**Background:**

Bevacizumab, an anti-VEGF monoclonal antibody, has recently emerged as a new option for severe forms of hereditary hemorrhagic telangiectasia (HHT). Its utilization in this orphan disease has rapidly spread despite the lack of randomized trials and international guidelines. The objective of this study is to report the main clinical data (baseline characteristics, dose schedule, efficacy, adverse events and deaths) of HHT patients treated by intravenous bevacizumab in France.

**Methods:**

Retrospective observational study of HHT patients treated with bevacizumab for a severe form of the disease in the 14 centers of the French HHT network.

**Results:**

Forty-six patients (median age: 68 years) were treated between March 2009 and May 2015. Ten patients were treated for high output cardiac failure, 20 patients for severe hemorrhages and 16 for both indications. The standard protocol (6 infusions of 5mg/kg every 2 weeks) was initially used in 89% of the cases but diverse strategies were subsequently applied. A clinical improvement was noted by the referent physician for 74% of the patients with a median effect’s duration of 6 months. Wound healing complications led to 2 amputations. Arthralgia/arthritis and arterial hypertension occurred in 5 patients each. One third of the patients were dead at the time of the final update, coherently with age and the poor prognosis of these highly symptomatic patients.

**Conclusion:**

Intravenous bevacizumab seems to provide a clinical benefice in severe HHT patients. Precautions concerning wound healing and vascular pathologies must be respected. Prospective double blinded versus placebo trials are needed.

## Introduction

Hereditary Hemorrhagic Telangiectasia (HHT) is a worldwide vascular genopathy with an incidence rate estimated from 1/5000 to 1/8000. This autosomic dominant disorder is caused by the haplo-insufficiency of at least 3 genes (*ENG*, *ALK1* and *SMAD4*) belonging to the BMP/SMAD signaling pathway. Data suggest that it results in an imbalanced state between pro- and anti-angiogenic factors, including a high level of Vascular Endothelial Growth Factor (VEGF), leading to dysregulation of the neo-angiogenesis [1]. The main clinical features are telangiectasia of the face, the fingers and the nasal mucosa, which are often complicated by recurrent epistaxis and anemia. Telangiectasia less frequently affect the gastro-intestinal (GI) tract and can cause occult bleedings. Larger arterio-venous malformations (AVMs) can occur, affecting the lung (with a risk of stroke or cerebral abscess), the liver (with a risk of high-output cardiac failure (HCF)), or the brain (with a risk of intracranial hemorrhage) [2].

Bevacizumab is an anti-VEGF monoclonal humanized antibody designed to inhibit the tumor induced neo-angiogenesis. Its clinical use is authorized since 2005 as an adjuvant treatment for many kinds of metastatic or locally advanced solid cancers [3]. In 2006 then 2008, its administration to HHT patients with a concurrent oncologic condition has been associated to a dramatic improvement of epistaxis and HCF [4,5]. Therefore, an open label phase 2 single arm trial has been conducted in France in 2009 in 25 patients with a symptomatic liver involvement. A significant decrease of high cardiac output and epistaxis duration has been demonstrated [6]. Since this date, bevacizumab has been used to treat severe HHT-related symptoms.

The aim of our study is to describe the use of bevacizumab through the French HHT network with particular considerations for efficacy, adverse effects and death causes in this specific orphan disease.

## Patients and methods

### Data collection

Cases were defined as adult patients with definite HHT diagnosis according to the Curaçao criteria and having already received at least one intra-venous injection of bevacizumab. Regular calls for cases have been made in the centers of the French HHT network between March 1st, 2009 and May 30th, 2015. A standardized form was sent to the physician in charge of the patient as soon as the case was notified. This form was designed to anonymously collect data on the indication of bevacizumab, demographic parameters, HHT diagnosis criteria and related symptoms, concomitant conditions and treatment, including dose schedule, adverse events and global efficacy.

Each case was classified in a group according to the therapeutic goal: attenuating the high-output heart failure due to hepatic AVM (HCF group), reducing the transfusion needs or the number of life-threatening hemorrhages by epistaxis or GI bleeding (Severe Hemorrhage (SH) group) or both (HCF+SH group). Standard protocol was considered as 6 administrations of 5 mg/kg of bevacizumab, each spaced by 14 days. Re-treatment’s procedure was defined by a new administration sequence in case of symptom recurrence after the end of the initial treatment. Maintenance therapy was defined by systematic and planned administrations after the initial treatment, independently from the intensity of HHT-related symptoms. To objectively assess the treatment efficacy, physicians were requested to detail, if available, NYHA class of dyspnea, cardiac index, hemoglobin level, transfusion frequency and epistaxis duration, before and after treatment. A global judgment of the clinical efficacy (improvement, stability, aggravation) was also systematically asked to the physician after having reviewed their files with patient’s statements, physical signs, laboratory results, imaging and associated treatment. A minimum period of three months after the first injection was required to establish this assessment. All clinically relevant events occurring after the first injection were considered as adverse events and registered, whatever the time interval between the treatment and the event. Exceptions were made for GI bleeding and epistaxis, considered as usual HHT hemorrhagic events.

Once completed and returned, all the forms were reviewed by AG or SR. Complementary data were directly asked to the physician for clarification if required. In case of death, the main cause and the associated circumstances were discussed between the referent physician and AG or SR.

The current status (ongoing treatment, adverse event, death) of all included patients was updated between July and August, 2015.

### Statistical analysis

Patients and treatments characteristics are presented in tables. Quantitative variables are described with median, minimum and maximum values. Categorical variables are given in absolute number and percentage. Statistical comparisons between HCF, SH and HCF+SH groups were judged non pertinent given the objective and the size of the study. Potential predicting factors of efficacy were assessed by univariate models of logistic regression.

A specific R script was built to generate all the statistical data (R version 3.1.2 (2014-10-31)) [7]. This script is fully available at https://doi.org/10.17026/dans-z7r-d74x. A p-value below 0.05 was considered as significant.

### Ethics statement

Patients having medical care through the French HHT network give a written consent for epidemiological studies prior to be included in the national HHT database (CIROCO). This computerized database is registered and approved by the French National Committee for Information Technology and Freedom (CNIL).

All patients were informed by their referent physician of the benefits and the risks potentially associated with bevacizumab and gave informed consent. In accordance to the French law (Loi française n° 2004–806 du 9 août 2004 relative à la politique de santé publique et ses décrets d’application), specific authorization by an ethic committee is not mandatory for a retrospective non-interventional protocol.

## Results

### Patient’s characteristics

Forty-six patients received intra-venous bevacizumab between March 2009 and May 2015. All of them were included in the study and analyzed. The median duration of the treatment was 106 days, corresponding to a total exposition of 51 patient-years.

With a median age of 68 years and a well-balanced sex ratio (48% male), the cohort was characterized by a large majority of mutated ALK1 carriers (80%), with hepatic (89%) and digestive (63%) involvements. One patient had received a hepatic transplant for HHT-related disease 15 years before. The most common comorbidities were atrial fibrillation (24%) and arterial hypertension (22%). About one quarter (24%) of the patients had previously experienced at least one venous thrombo-embolic event and a similar proportion had a history of severe sepsis (Table 1).

**Table 1:** Patient’s characteristics before treatment (n = 46).

Age (years), (median, (min-max))	68	(35–83)
Gender (male) (number, (%))	22	(48)
HHT mutation (number, (%))		
ENG	4	(9)
ALK1	37	(80)
SMAD4	0	(0)
No mutation found	5	(11)
Epistaxis (number, (%))	44	(96)
Mucocutaneous telangiectasia (number, (%))	45	(98)
Digestive telangiectasia (number/number screened, (%))	24/38	(63)
Hepatic AVM (number/number screened, (%))	41/46	(89)
Pulmonary AVM (number/number screened, (%))	13/46	(28)
Cerebral AVM (number/number screened, (%))	1/36	(3)
Liver transplantation (number, (%))	1	(2)
Associated conditions		
Hypertension (number, (%)	10	(22)
Atrial fibrillation (number, (%)	11	(24)
Cardiopathy^a^ (number, (%)	6	(13)
Hypothyroidism (number, (%)	4	(9)
Other^b^ (number, (%)	10	(22)
Significant medical histories		
Venous thrombo-embolic disease (number, (%))	11	(24)
Severe sepsis (number, (%))	13	(28)
Cancer^c^ (number, (%))	6	(13)
Gastric ulcer (number, (%))	3	(7)
Other^d^ (number, (%))	8	(17)

AVM: arterio-venous malformation

^a^: 1alcoholic dilated cardiomyopathy, 2 ischemic cardiomyopathies, 3 valvular cardiomyopathies

^b^: 1 myelodysplasia, 1 chronic C hepatitis, 1 atopic disease, 1 chronic renal failure, 1 adrenal insufficiency, 1 meningioma, 1 arthrosis, 1 intellectual deficiency, 1 benign prostatic hyperplasia, 1 sleep apnea

^c^: 1 breast cancer, 3 renal carcinomas, 2 skin basal cell carcinomas

^d^: 2 depressions, 1 leg ulcer, 2 alcohol diseases, 3 asthmas

### Bevacizumab use

Table 2 describes bevacizumab use. Among the 10 patients (22%) treated only for symptomatic high output cardiac failure (HCF), 8 (80%) had a class III or IV NYHA dyspnea at the beginning of the treatment. The median cardiac index was 5.2 l/min/m2, after exclusion of patients with atrial fibrillation (cardiac index is not reliable in this situation). Twenty patients (44%) who were treated exclusively for severe hemorrhages were receiving a median of 3 packed red blood cells (PRBC) per month before treatment. One of them was affected by rare but massive and life threatening epistaxis with subnormal hemoglobin level between the episodes (113 g/l just before the treatment’s start). Sixteen patients (35%) were treated concomitantly for both indications.

**Table 2:** Initial severity of disease, modalities of use, efficacy and safety of bevacizumab.

	HCF	SH	HCF+SH	Total
Number of patients	10	20	16	46
**Initial severity**				
PRBC per month (n, (med-min/max))	-	3 (1–6)	2,75 (1–5)	
Hemoglobin level (g/l, (med-min/max))	-	78 (30–113)	85 (65–144)	
III or IV NYHA class of dyspnea (number, (%))	8 (80)	-	6 (38)	
Cardiac index^a^ (l/min/m2, (med-min/max))	5,2 (3,5–11,1)	-	5,4 (4,3–8,2)	
**Modality of use**				
Standard protocol use (number, (%))	10 (100)	16 (80)	15 (94)	41 (89)
Maintenance therapy (number, (%))	1 (10)	8 (40)	5 (31)	14 (30)
Re-treatment (number, (%))	1 (10)	6 (30)	4 (25)	11 (24)
Duration of treatment (days, (med-min/max))	75 (17–2241)	235 (17–1499)	115 (32–1589)	106 (17–2241)
Liver transplantation after treatment (number, (%))	3 (30)	0 (0)	0 (0)	3 (7)
**Efficacy**				
Improvement (number, (%))	6 (60)	16 (80)	12 (75)	34 (74)
Improvement’s duration (months, (med-min/max))	5 (5–5)	6 (1,5–12)	6 (2–20)	6 (1–20)
**Adverse events**				
At least one adverse event (number, (%))	5 (50)	16 (80)	9 (63)	30 (65)
Infection (number, (%))	2 (20)	4 (20)	4 (25)	10 (22)
Hemorrhagic event^b^ (number, (%))	1 (10)	0 (0)	0 (0)	1 (2)
Arthralgia/arthritis (number, (%))	0 (0)	4 (20)	1 (6)	5 (11)
Hypertension (number, (%))	1 (10)	3 (15)	1 (6)	5 (11)
Cardiac failure (number, (%))	1 (10)	2 (10)	1 (6)	4 (9)
Wound concern (number, (%))	0 (0)	2 (10)	1 (6)	3 (7)
Ischemic event^c^ (number, (%))	1 (10)	0 (0)	1 (6)	2 (4)
Efficacy loss (number, (%))	0 (0)	2 (10)	0 (0)	2 (4)
Proteinuria (number, (%))	0 (0)	0 (0)	0 (0)	0 (0)
Rebound effect at treatment cessation (number, (%))	0 (0)	0 (0)	0 (0)	0 (0)
Other^d^ (number, (%))	2 (20)	12 (60)	4 (25)	18 (39)
Death during treatment’s period^e^ (number, (%))	1 (10)	1 (5)	1 (6)	3 (7)

HCF: High-output Cardiac Failure

SH: Severe Hemorrhage

PRBC: packed red blood cells

^a^: patients with known atrial fibrillation were excluded (unreliability of the echographic assessment)

^b^: Except usual HHT hemorrhagic events (GI bleeding or epistaxis)

^c^: 1 ischemic cholangitis, 1 mesenteric thrombosis

^d^: 3 asthenias, 3 nonspecific pain, 2 neutropenia, 1 muscular weakness, 1 hypocalcemia, 2 depressions, 2 small cell lymphoma, 1 hip fracture, 1 gastritis, 1 gastro-esophageal reflux, 1 minor allergic reaction

^e^: treatment period is defined as the time interval between the first injection and six months after the last injection

The standard protocol was the most often used (89%) for initial treatment. A treatment continuation was decided for about the half of the cohort (54%) (re-treatment or maintenance therapy). Twelve patients were treated for more than one year. Four patients received bevacizumab injections during more than 4 years.

Three patients underwent orthotopic liver transplantation after the bevacizumab treatment. They were 46, 53 and 63 years old, all treated for HCF and considered as improved by bevacizumab. One of them had experienced an adverse event (haemoperitoneum) leading to the discontinuation of the treatment. The time between the last injection of bevacizumab and the surgical procedure was 11, 15 and 17 months. No specific concerns were raised after the transplant procedure, especially concerning wound healing.

Objective measures of efficacy after treatment (NYHA class of dyspnea, cardiac index, hemoglobin level, transfusion frequency and epistaxis duration) were very heterogeneous in terms of modalities and timing of evaluation. In some cases, data were missing. For these reasons, we were not able to adequately perform statistical analysis of the objective efficacy parameters. The clinical appreciation of efficacy by the referent physician was obtained for all cases. Improvement of the HHT-related symptoms was noted in 74% of the whole cohort. The median duration of efficacy was 6 months (minimum: 1 month, maximum: 20 months). In univariate, no patient characteristics were found to be significantly associated with bevacizumab efficacy. No improvement was observed in both cases of a twin’s pair, treated at an interval of 15 months.

The most frequent adverse event was infections (22%) including 3 pneumonias, 1 bacteremia, 1 osteitis, 1 urinary tract infection, 1 epididymitis, 1 ophthalmic infection, 1 herpes zoster, 1 sepsis of undetermined origin. Arterial hypertension and rheumatologic symptoms were less frequent (11% each). Wound-healing complications occurred in 3 patients, 2 of them underwent leg amputation and the third developed of severe bedsores. Loss of efficacy under prolonged therapy (maintenance or re-treatment) was reported for 2 patients but no exacerbation of HHT-related symptoms was observed after treatment cessation. No case of proteinuria was notified. Fifteen (33%) patients died during the follow-up.

Details on the death cases having occurred during the follow-up are reported in S1 Table. Seven deaths were related to heart failure, 4 to sepsis and 2 to massive hemorrhages. Three deaths (2 sepsis, 1 ischemic cholangitis) occurred within 6 months after the last injection. Most of these 15 patients were considered as bevacizumab non-responders or had stopped it after an adverse event.

## Discussion

### Efficacy

With an improvement of 74% of the whole cohort, our study strongly suggests a clinical benefit of bevacizumab for HHT patients. This result is only based on physicians’ judgments since the objective parameters of efficacy were unfortunately not exploitable (different timing and modalities of evaluation, missing data). The overall assessment of an expert physician is considered as a reliable and relevant scoring system in many complex diseases. By example, the PGA (physician’s global assessment) in lupus and the IGA (Investigator global assessment) in atopic dermatitis are well-recognized scores based on this principle [8–10].

Bevacizumab seems to be as effective in severe hemorrhage as in HCF. This is consistent with the central role of the VEGF dysregulation in HHT [11].

Bevacizumab appears to have an only suspensive effect. All patients have experienced symptoms recurrence after the discontinuation of the treatment, but after a large range of time (1 to 20 months). The same phenomenon has recently been reported in the long term study of the patients included in the French open label trial [12] and is coherent with the pharmacological effects of the drug [13]. Prolonged therapy (re-treatment or maintenance) was more frequent in the SH group (70% vs 20% and 56% for HCF and HCF+SH), potentially because of a clinically more obvious benefit. Loss of efficacy is rare, and no rebound effect after treatment cessation has been observed.

We observed that about one quarter of the patients did not exhibit a clinical improvement. This is higher than the 17% reported by Dupuis-Girod et al. in a prospective trial [6] but our cohort is composed of non-selected patients with concomitant conditions encountered in the daily practice. No patient’s characteristic at the baseline seems to be predictive to a bevacizumab response. The lack of bevacizumab efficacy in the twin’s pair argues for a role of the genetic background. Polymorphisms and genetic modifiers are likely to explain the high variability of HHT expression among carriers of the same mutation [14,15]. They could also modulate the patient’s sensibility to bevacizumab. Further studies on larger populations should be conducted to identify such predictive factors and guide therapeutic choices. Until then, the only possibility is to initiate treatment for each patient in order to test his individual responsiveness.

Orthotopic liver transplant remains the only curative option in case of severe hepatic involvement. Three of our patients were treated by bevacizumab prior to the transplant procedure. No specific problems were reported but a long delay between the last injection and the surgery was respected. Bevacizumab seems to be able to improve the pre-transplant clinical status and could be a valuable option for frail candidates to surgery. It also involves a risk of missing the “transplant window” for patients under 65 years and the decision to treat with bevacizumab must be carefully considered in this context [16].

The study was not designed to properly analyze the medico-economic impact of bevacizumab. Nevertheless, for the most highly responsive patients of the cohort, this treatment is an obvious cost-effective option given the reduction of overall hospital care costs related to acute hemorrhagic events and heart failure. This economic benefit has also been suggested by others studies [17,18] and is an important point to assess in the future.

### Adverse events

One important contribution of our study is to provide a global view of the adverse events associated with bevacizumab in this chronic and non-oncologic context.

Blood pressure elevation has concerned 5 patients (11%). It is an expected side effect of bevacizumab and can be most of time easily managed [19]. Arthralgia or arthritis has been reported in 5 (11%) cases here, although it is not a well-known side-effect of bevacizumab. It could correspond to an attenuated form of serum sickness related to immune complexes including bevacizumab as described with other monoclonal antibodies [20]. Other mechanisms could be hypothesized, like hypoxia induced by synovial inflammation due the anti-VEGF effect [21,22] or a complication of severe hypophosphatemia related to intravenous iron administration [23], frequently used in this population. Next cases should be more precisely documented to better understand the underlying physiopathology. The high frequency of infections and heart failure are more probably related to the natural evolution of HHT [24,25] but have also been described with bevacizumab [26,27]. No case of venous thromboembolism has been diagnosed during the study, but it has been recently reported by Maestraggi [28]. Surprisingly, no proteinuria has been reported while it is one of the major concern for type 2 neurofibromatosis’s patients treated by bevacizumab [29,30]. This adverse effect is reported with a median time under treatment longer than 2 years, which is the case for only 6 patients in the present study. It should regularly be checked, especially in case of long term treatment.

Among the various adverse events reported here, the wound healing complications were highly problematic and have led to two cases of lower limb amputation. This process appears to be importantly disturbed by bevacizumab and special attention to skin wound and surgery should be kept in mind. In the oncologic context, it is recommended to stop bevacizumab 6 to 8 weeks before the surgery and to restart it at least 4 weeks after and only if the wound is totally healed [31]. In the same way, unstable or severe arteriopathy as well as recent thrombotic events are highly risked situations and should reasonably lead to delay or refuse this treatment [32–34].

In this cohort, 33% of patients died during the follow up, essentially from usual HHT complications: end-stage heart failure, sepsis and cataclysmic hemorrhage. It has occurred either by unresponsive patients or after a long time after treatment cessation (up to 55 months). Mortality studies in HHT patients are scarce. In UK, diagnosis of HHT is associated with a 3-years reduction in median age at death [35]. In contrast, Kjeldsen et al [36] have shown a high mortality rate (20 of 28 patients on a 21 years’ period) in a group of patients with severe HHT-related symptoms characterized by “daily or weekly episodes of severe bleeding leading to severe anemia and transfusions, hospitalization”. Most of our patients could have been classified in this group and this severe prognosis constitutes an obvious recruitment bias explaining the observed mortality rate. These observations argue for proposing bevacizumab earlier in the disease’s natural history, to less frail subjects. Two treatment thresholds could be considered: a prolonged state of transfusion dependency or the persistence of heart failure symptoms after cardiologic treatment optimization.

### Limitations of the study

Our work suffers from limitations related to its retrospective, non-interventional and open design. The retrospective data collection and the diversity of participating centers have not allowed us to obtain standardized objective parameters for the efficacy assessment. Nevertheless, we think that our data honestly reflect the daily practice and provide valuable information to apprehend the risk/benefit ratio of this new drug. It constitutes a preliminary step to conducting a large, prospective, and randomized versus placebo trial, which is deeply needed.

The lack of consensual modalities of bevacizumab use, especially for long term treatments, is another concern. It has generated a high level of heterogeneity in our data. Most of physicians have initially followed the "standard protocol" of 6 injections of 5mg/kg, by analogy with the oncological use and the prospective trial [6]. Other protocols with lower doses have also been proposed [37,38] and could be more appropriate in some cases, notably for frail patients. Concerning the subsequent treatment (maintenance or retreatment), a pharmacological study based on a mathematical model suggested a systematic monthly injection [39]. A retreatment strategy individually adapted to the clinical needs has been applied for 24% of the cohort. This strategy could be pertinent but requires to be sustained by further clinical and biopharmaceutical investigations.

## Conclusion

**Fig 1:**
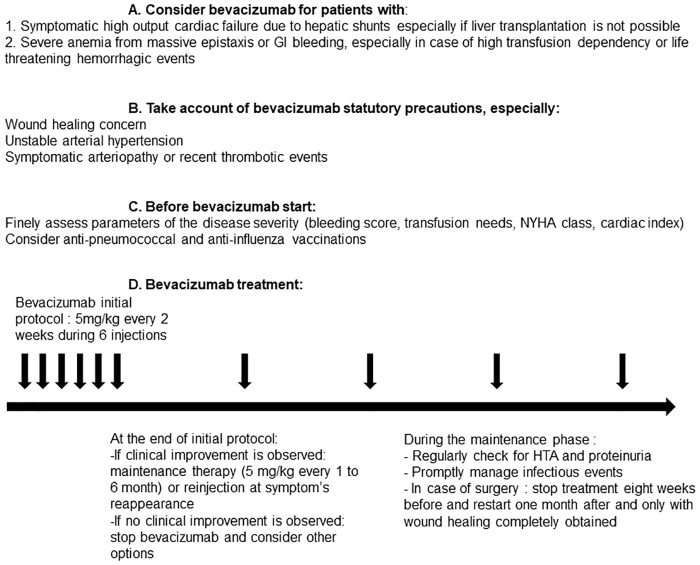
Recommendations for the use of intra-venous bevacizumab in HHT patients.

In the field of rare diseases, therapeutic innovations are rare and challenging events. This multicenter retrospective study of 46 patients is, to our knowledge, the most important retrospective cohort of HHT patients treated by intra-venous bevacizumab. It supports a clinically pertinent therapeutic effect for highly symptomatic patients. Wound’s healing complications are a major limitation and should be considered as a contraindication. This collective experience leads us to provide some basic guidelines for the French “temporary use recommendations” of bevacizumab for HHT patients, summarized in Fig 1. A prospective double blinded versus placebo study for intra-venous bevacizumab in HHT is needed.

## Supporting information

S1 Table Characteristics of the cohort’s patients who died during the follow up. (DOCX)
