# Supplementary material for: Intra-venous bevacizumab in hereditary hemorrhagic telangiectasia (HHT): A retrospective study of 46 patients
Source: PLoS One. 2017 Nov 30;12(11):e0188943. doi: 10.1371/journal.pone.0188943 (PMC5708634; doi:10.1371/journal.pone.0188943)
Supplement: S1 Table — (DOCX) [file pone.0188943.s001.docx]

|  | Age (years) | Gender | Mutation | Pre-existing condition (s) | Main indication | Efficacy^a^ | Time last injection/ death (days) | Total dose received (mg/kg) | Serious adverse event(s) | Maintenance therapy | Cause of death |
| --- | --- | --- | --- | --- | --- | --- | --- | --- | --- | --- | --- |
| Death during the first 6 months after the last injection | | | | | | | | | | | |
| 1 | 78 | M | ENG | Ischemic cardiopathy, VTED | SH | No | 65 | 15 | Pneumonia, bacteriemia | No | Sepsis with HCF |
| 2 | 61 | F | Unknown | Meningioma | HCF | No | 72 | 10 | Ischemic cholangitis, pneumonia, gastritis, depression | No | Ischemic cholangitis |
| 3 | 64 | F | Unknown | VTED | HCF+ SH | No | 129 | 30 | GI bleeding | No | Sepsis |
| Death between 6 and 12 months after the last injection | | | | | | | | | | | |
| 4 | 74 | M | ALK1 | HTA, AF, liver transplantation | HCF+ SH | Yes | 229 | 10 | Wound concern | No | Multi-organic failure |
| 5 | 76 | M | ALK1 | 0 | SH | Yes | 286 | 35 | Arthralgia | No | Massive hemorrhage |
| 6 | 53 | F | ALK1 | AF | HCF | Yes | 346 | 10 | Haemoperitoneum | No | HCF after hepatic graft |
| Death after the first year after the last injection | | | | | | | | | | | |
| 7 | 35 | F | ALK1 |  | SH | Yes | 386 | 90 | Transient HCF | Yes | HCF |
| 8 | 70 | F | ALK1 | Hypothyroidism, VTED | SH | Yes | 448 | 45 | Asthenia, nonspecific pain, efficacy loss | No | Blood inhalation |
| 9^b^ | 73 | F | ALK1 | AF | HCF+ SH | No | 516 | 30 | 0 | No | Hepatic encephalopathy |
| 10 | 67 | M | ALK1 | Alcohol disease | HCF+ SH | Yes | 548 | 60 | 0 | No | Pneumonia + ascitis |
| 11 | 75 | M | ALK1 | AF, arthrosis | HCF | No | 570 | 30 | Epidydimitis | No | HCF |
| 12 | 57 | F | ALK1 | AF, valvular cardiopathy | HCF | Yes | 699 | 30 | 0 | No | Heart failure |
| 13 | 73 | F | ALK1 | AF | HCF+ SH | Yes | 1107 | 30 | Stroke^c^ | No | Stroke |
| 14^b^ | 72 | F | ALK1 | HTA | HCF+ SH | No | 1388 | 30 | Urinary tract infection | No | HCF |
| 15 | 73 | F | ALK1 | AF, hypothyroidism | HCF+ SH | Yes | 1606 | 30 | Hip fracture | No | Pneumonia |

Gender: M: male, F: female

Main indication: HCF: High-output Cardiac Failure, SH: Severe Hemorrhage

Efficacy of bevazicumab (physician global assessment): 0: None, +: moderate improvement, ++: important improvement

AF: atrial fibrillation

VTED: venous thrombo-embolic disease

^a^: overall efficacy as evaluated by the local physician

^b^: twins

^c^: 36 months after treatment
